# In vivo serotonin 1A receptor distribution in treatment-resistant depression

**DOI:** 10.1038/s41398-025-03406-3

**Published:** 2025-06-03

**Authors:** Matej Murgaš, Christian Milz, Peter Stöhrmann, Jakob Unterholzner, Lukas Nics, Georg S. Kranz, Andreas Hahn, Marcus Hacker, Siegfried Kasper, Rupert Lanzenberger, Godber M. Godbersen

**Affiliations:** 1 Department of Psychiatry and Psychotherapy, Medical University of Vienna, Vienna, Austria; 2 Comprehensive Center for Clinical Neurosciences and Mental Health, Medical University of Vienna, Vienna, Austria; 3 Department of Biomedical Imaging and Image-guided Therapy, Division of Nuclear Medicine, Medical University of Vienna, Vienna, Austria; 4 Department of Rehabilitation Sciences, The Hong Kong Polytechnic University, Hung Hom, Hong Kong; 5 Center for Brain Research, Department of Molecular Neuroscience, Medical University of Vienna, Vienna, Austria

**Keywords:** Depression, Neuroscience

## Abstract

Major depressive disorder (MDD) ranks among the leading causes of disability worldwide. An additional burden arises from treatment-resistance, defined by a lack of response to two or more adequate pharmacotherapeutic treatment trials. Unlike in MDD, where the serotonin 1A receptor subtype (5-HT_1A_) has commonly been used to study pathophysiological alterations, treatment-resistant depression (TRD) subjects represent a less investigated cohort. In this cross-sectional study, 5-HT_1A_ receptor binding was assessed in 33 subjects with TRD with stable medication and 44 healthy control (HC) subjects. Positron emission tomography scans with the radioligand [*carbonyl*-^11^C]WAY-100635 were acquired and 5-HT_1A_ receptor nondisplaceable binding potential (BP_ND_) was quantified using the multilinear reference tissue model 2. Regional BP_ND_ in amygdala, anterior cingulate cortex, hippocampus, insula, orbitofrontal cortex, dorsal raphe nucleus and median raphe nucleus was assessed using a multivariate analysis of covariance (MANCOVA). The MANCOVA showed a significant effect of group (F = 3.349, *p* < 0.05) and sex (F = 2.428, *p* < 0.05). The subsequent pairwise comparison revealed a lower BP_ND_ by 17.45% in the TRD group in the dorsal raphe nucleus (mean difference ± SE = −0.59 ± 0.24, *p* < 0.05) and by 18.39% in the median raphe nucleus (mean difference ± SE = −0.71 ± 0.30, *p* < 0.05). Our results extend previously reported alterations of 5-HT_1A_ receptor distribution in non-resistant depression to TRD. Ultimately, this knowledge may contribute to clarifying the role of serotonin and help to address the urgent issue of treatment resistance in depression.

## Introduction

Major depressive disorder (MDD) is one of the leading causes of disability worldwide [1]. A particular challenge arises from depressive episodes that show insufficient response to first-line treatment. The lack of response to two or more adequate psychopharmacological treatment trials is commonly referred to as treatment-resistant depression (TRD). As approved treatment options appear to be ineffective in TRD, one might also expect to find differences on a pharmacological level. With regard to the considerable economic and personal consequences of treatment-resistance, understanding the biological characteristics of this form of depression is of particular relevance.

Current models for the pathophysiology of depression consider various biopsychosocial factors [2], including alterations within the serotonergic system [3]. The serotonin 1A (5-HT_1A_) receptor is one of the most abundant serotonin receptors in the human brain and, thus, among the most researched subtypes. In the past, pathophysiological states and changes to its distribution in the cortex were connected to different psychiatric disorders, including MDD [4, 5]. Positron emission tomography (PET) has become a valuable modality for investigating pathophysiological molecular processes in psychiatric disorders in vivo. Previous studies have shown alterations of 5-HT_1A_ receptor binding potential in MDD patients across various brain regions, including the raphe nuclei [5, 6]. However, there have been inconsistencies regarding the direction of these alterations [7]. On the one hand, a reduction in 5-HT_1A_ receptor availability across different limbic and cortical regions, mesiotemporal cortex and raphe nuclei was demonstrated in various cohorts of MDD patients (including in non-remitters) [6, 8–10]. However, on the other hand, convincing publications report increases in cortical and subcortical regions when comparing MDD patients with healthy controls [7, 11]. These discrepancies were reconciled when replication studies concluded that heterogeneous outcome measures and quantification methods were the most plausible origin of the divergent findings [11, 12]. Still, care needs to be taken when comparing and interpreting these results.

Despite the interest in various different MDD cohorts, only a few studies have already described treatment effects on 5-HT_1A_ receptor binding potential in treatment-resistant depression. Previous work from our group suggests that electroconvulsive therapy (ECT) leads to decreased binding in 5-HT_1A_ receptor-rich regions of the amygdala, anterior cingulate cortex, hippocampus, orbitofrontal cortex and insula [13]. Moreover, in a recent study, we report that increased 5-HT_1A_ receptor availability in the dorsolateral prefrontal cortex, induced by transcranial magnetic stimulation (TMS), may lead to a decrease in depression severity in patients with TRD [14]. However, a comparison of 5-HT_1A_ receptor binding between healthy individuals and patients with TRD prior to ECT or TMS has not been published yet.

In this work, we assess differences in 5-HT_1A_ receptor binding potential between treatment-resistant depressive patients at stable medication and healthy control subjects. In particular, we aim to investigate previously implicated high-binding regions (including the anterior cingulate cortex, amygdala, hippocampus, insula, orbitofrontal cortex and dorsal and median raphe nuclei) using in vivo positron emission tomography with the high-affinity radioligand [*carbonyl*-^11^C]WAY-100635. In line with previous work on major depressive disorder and associated changes to the serotonergic system, we hypothesize that there are also alterations in serotonin 1A receptor binding potential in patients with TRD.

## Materials and methods

### Subjects and study design

The dataset represents a collection of published studies alongside previously unpublished data. The data of healthy control subjects (HC) were taken from Lanzenberger et al. [15] and Baldinger et al. [16] (registered at the International Standard Randomized Controlled Trial Number Register as ISRCTN30885829). The treatment-resistant depression group data was adopted from Lanzenberger et al. [13], Murgaš et al. [14] and complemented by an additional set of unpublished data collected as a part of the latter study (registered at ClinicalTrials.gov as NCT02810717). For details on demographic information, see Table 1 in the results section and Supplementary Table 1.

**Table 1:** Demographic information on the subject cohort.

	Subjects with TRD	HC subjects
N	33	44
Sex F/M	22/11	15/29
Age (mean ± SD)	40.5 ± 11.4	25.2 ± 3.5
HAMD (mean ± SD)	24.1 ± 5.3	N/A

Demographic information on treatment-resistant depressive (*TRD*) patients and healthy control (*HC*) subjects after exclusion of two outliers. *F* = female, *M* = male, *HAMD* = 17-item Hamilton Rating Scale for Depression.

Routine medical examinations, including laboratory measurements, general physical and neurological status and an electrocardiogram, were performed to screen subjects for physical abnormalities. Moreover, clinical interviews by experienced psychiatrists ensured the mental health of the HC. Each TRD subject was carefully screened by a trained psychiatrist using SCID IV (Structural Clinical Interview for DSM IV Diagnosis) and included only if they had already undergone at least two adequate treatment trials with antidepressants and were currently on antidepressant medication. The TRD subjects from Murgaš et al. [14], as well as the newly included subjects, fulfilled the criteria for a single or recurrent major depressive episode having a score ≥ 18 on the 17-item Hamilton Rating Scale for Depression (HAMD) at the screening visit. Lanzenberger et al. [13] included TRD subjects with HAMD ≥ 23 at the screening visit. Subjects with current or past symptoms of mania, schizophrenia or schizoaffective disorder were not included in the studies. TRD subjects currently receiving medication targeting the 5-HT_1A_ directly, in particular aripiprazol, amitriptyline, buspirone, chlorpromazine, clozapine, nebivolol, nefazodone, pindolol, propranolol, quetiapine (>100 mg), risperidone, trazodone, triptans and ziprasidone, were excluded. Additionally, TRD subjects receiving mirtazapine from clinical trial NCT02810717 were not included. Additionally, the healthy volunteers in Baldinger et al. [16] did not exceed a daily consumption of the equivalent quantity of 20 g of alcohol or 10 cigarettes, while Lanzenberger et al. [15] included only male subjects. Only subjects with the available medication history were included in this study. Solely baseline PET measurements of the TRD subjects with no additional challenge or treatment were included. Subjects gave written informed consent at the screening visit. All procedures were performed according to the Declaration of Helsinki. All studies were approved by the Ethics Committee at the Medical University of Vienna (318/2002; 475/2011; 1761/2015).

### Neuroimaging

Imaging procedures for HC and TRD groups were similar, although details may vary. For each subject, a 90-min PET scan was acquired using a GE Advance PET scanner (General Electric Medical Systems, Milwaukee, Wisconsin) at the Department of Biomedical Imaging and Image-guided Therapy, Division of Nuclear Medicine, Medical University of Vienna, Austria. Acquisition in 3-D mode started with bolus infusion of the radioligand [*carbonyl*-^11^C]WAY-100635 with a dose of 4.6–5.4 MBq/kg. The radioligand [*carbonyl*-^11^C]WAY-100635 was prepared at the cyclotron unit of the PET Centre according to the previously described method [17]. A 5-min transmission scan in 2-dimensional mode with retractable ^68^Ge sources was used for attenuation correction. Images were reconstructed as a 128 × 128 matrix (35 slices) utilizing an iterative filtered back-projection algorithm with a spatial resolution of 4.36 mm full-width at a half-maximum 1 cm next to the centre of the field of view. Here, the number and the length of frames in the reconstructed dynamic PET images vary across the studies: Lanzenberger et al. [15] used 30 frames (15 × 60 s and 15 × 300 s), Baldinger et al. [16] used 50 frames (12 × 5 s, 6 × 10 s, 3 × 20 s, 6 × 30 s, 4 × 60 s, 5 × 120 s and 14 × 300 s). TRD subject groups from both studies [13, 14] were reconstructed to 51 frames (12 × 5 s, 6 × 10 s, 3 × 20 s, 6 × 30 s, 9 × 60 s and 15 × 300 s).

The following section refers to data acquisition and processing of unpublished baseline measurements of TRD subjects collected as a part of the study presented in Murgaš et al. [14]. For more details on the remaining data sets, please refer to the respective publications [13, 15, 16]. In addition to the PET scan, a structural T1-weighted magnetic resonance (MR) image was acquired for each subject. Subjects with TRD were scanned at a 3 T PRISMA MR Scanner (Siemens Medical, Erlangen, Germany) using the magnetization-prepared rapid gradient-echo sequence (TE/TR = 4.21/3000 ms, voxel size 1 × 1 × 1.1 mm^3^).

### Data processing and quantification

Each PET scan was corrected for tissue attenuation and scatter. Image pre-processing was performed using SPM12 (The Wellcome Centre for Neuroimaging, www.fil.ion.ucl.ac.uk) and MATLAB R2018b (The Mathworks Inc., Natick, MA, USA). Correction for head motion was applied, followed by the co-registration of dynamic PET images to the T1-weighted structural MR image. The structural image was then normalized to the Montreal Neurological Institute (MNI) space, generating a transformation matrix that was applied to the co-registered dynamic PET images as well.

Time activity curves (TACs) were extracted for selected regions of interest (ROIs) as described below and cerebellar white matter (CWM). The selected regions of interest have a high abundance of 5-HT_1A_ receptors and previously exhibited changes in 5-HT_1A_ receptor binding potential in TRD subjects suffering from major depressive disorder [13, 18]. These regions are the amygdala (AMY), anterior cingulate cortex (ACC), hippocampus (HIP), orbitofrontal cortex (OFC), insula (INS), dorsal raphe nucleus (DRN) and median raphe nucleus (MRN). Region-wise nondisplaceable binding potential (BP_ND_) was quantified with the multilinear reference tissue model 2 (MRTM2) with an individually fixed clearance rate of radioligand from reference tissue (k_2_’) [19]. Multilinear reference tissue model (MRTM) was utilized to estimate k_2_’ with insula as a receptor-rich region. In this paper, binding potential refers to BP_ND_ as it is described in the consensus on nomenclature [20]. Based on previous research [21], CWM was selected as the reference region, in contrast to earlier studies that used cerebellar gray matter [12]. The validity of CWM as a suitable reference region for [*carbonyl*-^11^C]WAY-100635 was also confirmed in a blocking experiment [11] as well as a post-mortem autoradiography study [22] showing the lowest 5-HT_1A_ receptor concentration in the cerebellar white matter. All quantification steps were done in PMOD, version 4.4 (PMOD Technologies LLC, https://www.pmod.com/web/). TACs were extracted using the Harvard-Oxford atlas as provided with FSL for AMY, ACC, HIP, OFC and INS. Regional values in MRN and DRN were extracted by manually placing a volume of interest for each subject around the voxel with local peak intensity in the summed normalized dynamic PET image. The volumes of interest for MRN and DRN were defined as a sphere with a radius of 3 mm comprising 14 voxels [23]. An in-house atlas [24] was used for CWM.

### Statistical analysis

Data harmonization was applied to correct possible bias introduced by data originating from different studies (e.g., different frame lengths). Here, we used the Matlab implementation of the ComBat toolbox [25–27] with age and sex as covariates for the healthy control group and using age, sex and HAMD for the TRD group, respectively. ComBat builds upon an empirical Bayes framework to reduce technical variability while at the same time preserving biological variability. This harmonization approach was shown to perform well with small sample sizes and unbalanced groups with regard to the biological covariates. Its design does not make any assumption on the nature of the imaging parameter of interest and is applicable to ROI-based as well as voxel-wise analyses. Before further analyses, the dataset was checked for univariate as well as multivariate outliers. Harmonized BP_ND_ values were assessed for univariate outliers for each combination of sex, group and region. For identification of multivariate outliers Mahalanobis distance was calculated for each subject, where one observation consists of the seven regional BP_ND_ values. Distances exceeding a critical χ^2^ value were excluded. To investigate the possible association between 5-HT_1A_ receptor binding potential and depression symptom severity, represented by HAMD scores, we used a multivariate analysis of covariance (MANCOVA). Here, only the TRD group was included, with the HAMD score taken close to the PET measurement and with age as an independent covariate and sex as a fixed factor. Next, the differences in 5-HT_1A_ receptor distribution between healthy control and TRD subjects were investigated. We utilized MANCOVA with the regional BP_ND_ values as dependent variables, group and sex as fixed factors, age as a covariate and interaction between group and sex. The exploratory post-hoc (not corrected for multiple comparisons) analyses were performed using pairwise comparison between group BP_ND_ corrected for age and sex or post-hoc Pearson correlation analysis between the significant covariate and BP_ND_ corrected for age and sex. Estimates of effect size are reported as partial eta squared (η^2^). Results with *p*-value < 0.05 were considered significant. Unless otherwise stated, all statistical analyses were performed in SPSS version 26 for Windows (SPSS Inc., Chicago, Illinois, USA; www.spss.com).

## Results

**Fig. 1:**
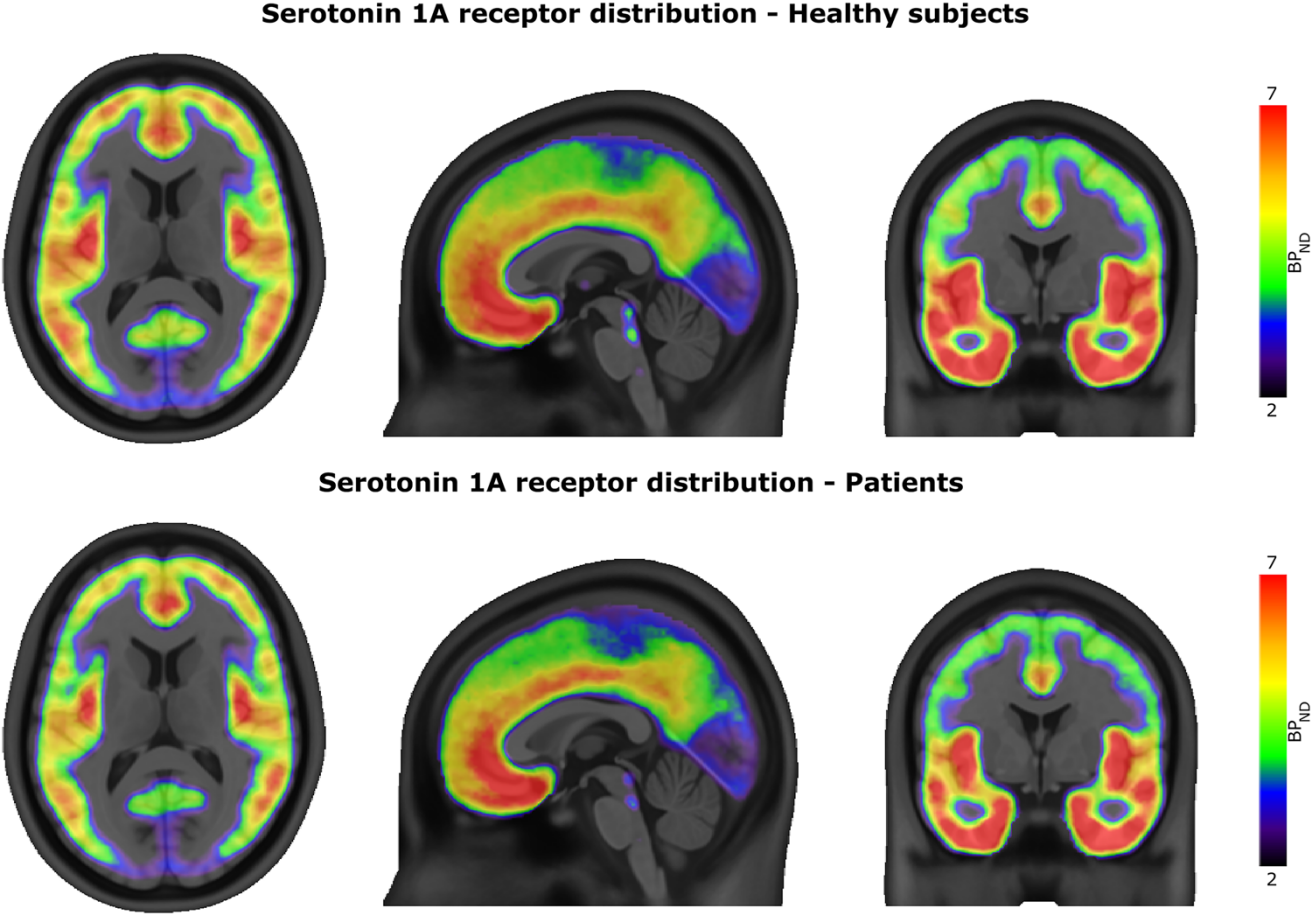
Volumetric representation of 5-HT_1A_ receptor binding potential. The tri-planar visualization (MNI coordinates x = 0 mm, y = 0 mm, z = 12 mm) of median serotonin 1A receptor distribution measured by PET with [*carbonyl*-^11^C]WAY-100635 radioligand. The comparison of nondisplaceable binding potential (BP_ND_) in the healthy subjects group (top row) and Patients with TRD group (bottom row) shows the highest difference in the regions of raphe nuclei (dorsal and median).

**Fig. 2:**
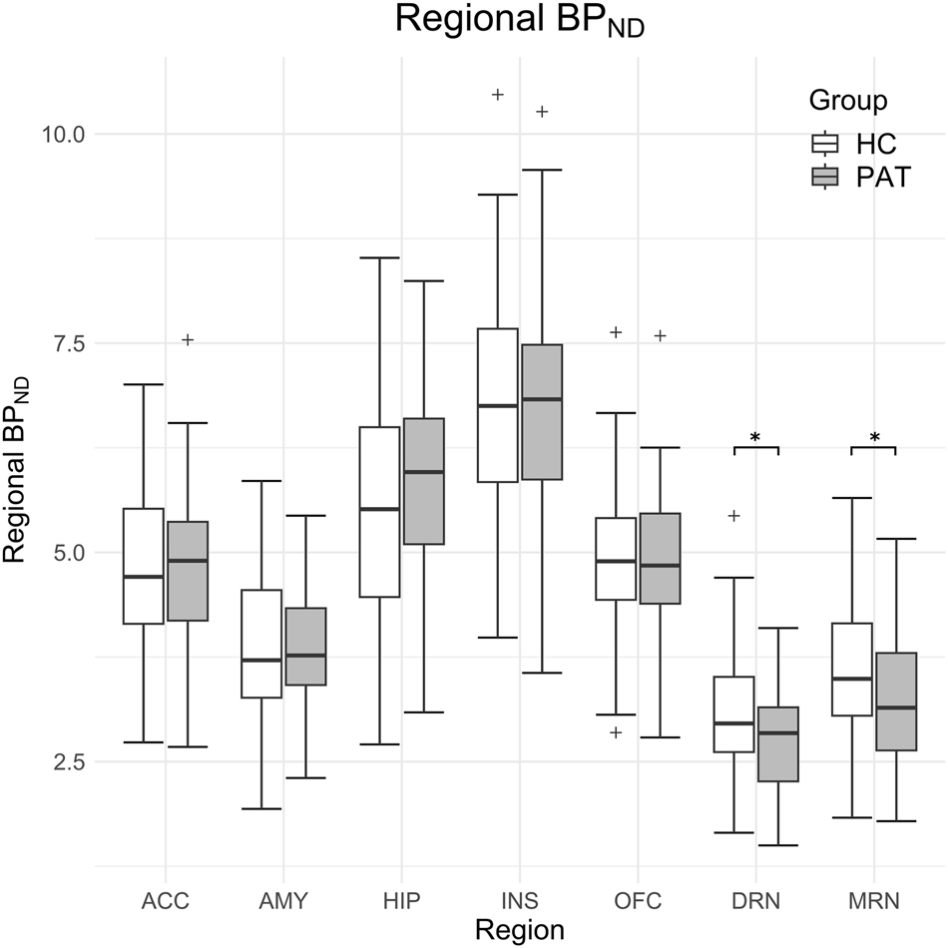
Comparison of region-wise 5-HT_1A_ BP_ND_ values in treatment-resistant depressive patients and healthy control subjects. The boxplot comparing 5-HT_1A_ BP_ND_ values between treatment-resistant depressive patients (PAT) and healthy control (HC) group regional An asterisk indicates significant differences between groups (*p* < 0.05). Regional BP_ND_ was corrected for sex and age. Middle lines represent median values. Values 1.5 times the inter-quartile range above/below the 75th/25th percentile are drawn as separate data points (outliers). Anterior cingulate cortex (ACC), amygdala (AMY), hippocampus (HIP), insula (INS), orbitofrontal cortex (OFC), dorsal raphe nucleus (DRN) and median raphe nucleus (MRN).

Data from 80 subjects were initially available for the comparison of serotonin 1A receptor distribution in selected regions. One healthy control subject was identified as a multivariate outlier based on Mahalanobis distance and one healthy control subject was identified as a univariate outlier by deviating more than three standard deviations from the mean. Both were excluded from all subsequent analyses. Ultimately, 44 HC subjects (mean age ± SD = 25.2 ± 3.5; 15 female) and 33 subjects with TRD (mean age ± SD = 40.5 ± 11.4; 22 female) were analyzed for this study (see Table 1). The BP_ND_ representing 5-HT_1A_ receptor distribution in both groups is shown in Fig. 1, complemented with estimated marginal means in Table 2 and BP_ND_ corrected for age and sex in Fig. 2.

**Table 2:** Comparison of 5-HT_1A_ BP_ND_ in healthy control and TRD subjects.

ROI	EMM of 5-HT_1A_ receptor BP_ND_			Pairwise Comparisons			
	HC	PAT	PD[%]	mean diff.	CI (95%)	Sig.	η^2^
ACC	4.66 ± 1.24	4.70 ± 1.28	0.84	0.04 ± 0.33	(−0.62, 0.70)	0.91	<0.001
AMY	3.83 ± 1.03	3.90 ± 1.06	1.86	0.07 ± 0.28	(−0.48, 0.62)	0.80	0.001
HIP	5.52 ± 1.68	5.93 ± 1.73	7.39	0.41 ± 0.45	(−0.49, 1.30)	0.37	0.011
INS	6.65 ± 1.73	6.71 ± 1.79	0.96	0.06 ± 0.46	(−0.86, 0.99)	0.89	<0.001
OFC	4.72 ± 1.19	4.75 ± 1.23	0.74	0.04 ± 0.32	(−0.60, 0.67)	0.91	<0.001
DRN	3.36 ± 0.91	2.78 ± 0.94	−17.45	−0.59 ± 0.24	(−1.07, −0.11)	<0.05	0.076
MRN	3.83 ± 1.11	3.13 ± 1.15	−18.39	−0.71 ± 0.30	(−1.30, −0.11)	<0.05	0.073

Estimated marginal means (*EMM*) and pairwise comparison of 5-HT_1A_ BP_ND_ between healthy control subjects (*HC*) and patients (*PAT*) group in anterior cingulate cortex (*ACC*), amygdala (*AMY*), hippocampus (*HIP*), insula (*INS*), orbitofrontal cortex (*OFC*), dorsal raphe nucleus (*DRN*) and median raphe nucleus (*MRN*). Marginal means are given as mean ± standard deviation and are corrected for sex and age. Percent Difference (*PD*) representing change of BP_ND_ in the individual regions is calculated as ${PD}=\left[{{BP}}_{{ND}}\left({PAT}\right)-{{BP}}_{{ND}}\left({HC}\right)\right]/{{BP}}_{{ND}}\left({HC}\right)\cdot 100 \%$. An exploratory post-hoc pairwise comparison is reported as the mean difference (±standard error) between the marginal mean ${{BP}}_{{ND}}\left({PAT}\right)$ and ${{BP}}_{{ND}}\left({HC}\right)$ with 95% confidence intervals (CI) and η^2^ representing the estimated effect size. The difference in marginal means was considered significant when *p* < 0.05.

The analysis of the effect of symptom severity in the TRD subject group showed a main effect of HAMD score on binding (F = 2.557, *p* < 0.05, η^2^ = 0.438) and sex (F = 2.830, *p* < 0.05, η^2^ = 0.463). The effect of age (F = 1.807, *p* = 0.13) was not significant. However, region-wise post-hoc correlation analysis between HAMD and BP_ND_ corrected for the sex and age did not show significant correlations (Supplementary Fig. 1) between 5-HT_1A_ BP_ND_ and HAMD in any investigated region. Similarly, the post-hoc pairwise comparison of male and female TRD subjects did not show significant differences in any particular region.

Using MANCOVA, with regional 5-HT_1A_ receptor BP_ND_ values as dependent variables to compare the TRD subjects and control group, showed a significant difference in receptor binding between the TRD group and the control group (F = 4.349, *p* < 0.05, η^2^ = 0.316, Fig. 2). Moreover, the effect of sex was significant as well (F = 2.428, *p* < 0.05, η^2^ = 0.205), while no effects of age (F = 1.776, *p* = 0.11) or interaction between group and sex (F = 1.440, *p* = 0.21) were unveiled. Exploratory tests of between-subject effects revealed a significant difference of BP_ND_ in the dorsal (F = 5.898, *p* < 0.05, η^2^ = 0.076) and median raphe nucleus (F = 4.508, *p* = < 0.05, η^2^ = 0.073) between TRD subjects and the control group but not in the remaining regions of interest. The estimated marginal means showed lower BP_ND_ in the TRD group (mean ± SD, DRN: 2.78 ± 0.94, MRN: 3.13 ± 1.15) than in the HC group (mean ± SD, DRN: 3.36 ± 0.91, MRN: 3.83 ± 1.11) in the raphe nuclei (see Table 2). In contrast, sex did not affect BP_ND_ significantly in any specific region (see Supplementary Table 2).

## Discussion

In this study, we investigated the differences in the 5-HT_1A_ receptor nondisplaceable binding potential between medicated subjects with TRD and healthy controls. A decrease in the presynaptic 5-HT_1A_ BP_ND_ in the dorsal and median raphe nuclei were found between TRD and control subjects. In addition, we unveiled an association between symptom severity assessed by HAMD and 5-HT_1A_ BP_ND_ in the TRD subject’s group. However, post-hoc analyses showed no significant region-wise effects of sex, age or symptom severity on receptor binding.

Post-hoc analysis of the difference in 5-HT_1A_ receptor binding between TRD and HC subjects showed a significant difference in some of the investigated regions. While both the dorsal and median raphe nuclei showed significantly lower BP_ND_, other regions exhibited no alterations to the binding. Similar to our results, a meta-analysis [6] has shown a significant reduction in 5-HT_1A_ receptor availability in the raphe nuclei. Studies [9, 28] using the whole cerebellum as a reference region reported these changes in MDD cohorts that were previously exposed to medication (yet medication free at least two weeks before the study). On the other hand, investigating discrepancies in BP_ND_ between antidepressant-naïve MDD and HC in the raphe nuclei using CWM as a reference region showed no changes at all [29]. Similarly, no statistically significant differences between antidepressant-naïve MDD, antidepressant-exposed MDD and HC were reported [30]. In line with our results, a post-mortem study suggested reduced 5-HT_1A_ binding capacity, representing a decrease in the number of receptors in DRN and MRN of depressed suicide victims when compared to healthy controls [31]. The lower 5-HT_1A_ receptor abundance in the raphe nuclei was suggested to be a result of the homeostatic response of the serotonin system [32]. In this context, the observed changes in raphe 5-HT_1A_ receptor BP_ND_ might indicate an imbalance in the serotonergic system. More specifically, these alterations lead to a decreased feedback inhibition mediated by the inhibitory 5-HT_1A_ autoreceptors in the raphe nuclei. The proposed mechanisms for reduced receptor binding are lower baseline expression, cell loss in the raphe nuclei or decreases in arborization and neuron size [8, 33]. Furthermore, concerning differences in localization and function of 5-HT_1A_ receptors, our results may indicate an imbalance between the raphe nuclei and the remaining ROIs. While inhibitory presynaptic 5-HT_1A_ autoreceptors with a regulatory role [23] are found in the raphe nuclei [8, 34], the other regions are rich in postsynaptic 5-HT_1A_ heteroreceptors [35]. However, previous research demonstrated a positive association between raphe binding and heteroreceptor binding [36].

Although the current understanding of the pathophysiology of depression certainly goes beyond serotonergic mechanisms alone [37, 38], antidepressant treatments targeting the serotonergic system have demonstrated clinical efficacy over decades [39]. With the raphe nuclei being a major source of serotonergic neurons, its pathophysiology was previously linked with depressive disorders [40, 41] but not specifically with TRD. Our results, therefore, substantiate the notion that perturbations in serotonergic neuromodulation indeed play a role in depression [42]. Specifically, regarding the question of whether TRD can be seen as a discrete biological entity from non-resistant depression [43], our findings point towards a similarity of both, at least regarding the lower 5-HT_1A_ autoreceptor BP_ND_ in the raphe nuclei [6]. However, a direct comparison between TRD and MDD cannot be readily drawn from our data, as these two groups were not compared directly in this work.

A valid question arising from our data concerns the potential influence of serotonergic medication on the results. In contrast to studies that include drug-naïve or not recently medicated MDD patients, here, we investigate a TRD subject cohort with constant concomitant medication. As per the definition of TRD, all TRD subjects underwent at least two different antidepressant treatments during the current depressive episode [44, 45] and were still under medication affecting the serotonergic system at the time of the PET scan. Although the effects of medication on TRD may theoretically manifest differently from MDD, we assume it to have a similar effect on 5-HT_1A_ receptor binding potential based on the congruent findings between the two conditions reported above and previous comparisons between treatment responders and nonresponders [7]. Moreover, prior research has shown that such treatment might only have region and substance-specific effects [46]. For example, long-term antidepressant treatment was suggested to reduce 5-HT_1A_ receptor density in limbic regions [47], but no significant effect was found in the DRN. Conversely, comparing medicated and drug-naïve MDD patients showed no statistically significant differences in BP_ND_ between these two groups, while both showed a lower abundance compared to a healthy control group [28, 48]. Moreover, a comparison of the BP_ND_ before and after the treatment with SSRI showed no difference 5-HT_1A_ receptor binding in the MDD subjects group [49]. In addition, in the data from Nash et al. [50], SSRI treatment, albeit in anxiety disorder, did not affect the reduced BP_ND_ in the raphe nuclei observed in both unmedicated and medicated participants. Furthermore, Parsey et al. [30] did not find statistically significant alterations in BP_ND_ between antidepressant naïve and medicated volunteers after two weeks of washout period. Ultimately, this does not contradict the idea of desensitization of 5-HT_1A_ autoreceptors after sustained SSRI treatment occurring through either internalization [8] or shifts from high- to low-affinity states. This is because 1) [*carbonyl-*^*11*^C]WAY-100635 as a 5-HT_1A_ antagonist binds to high as well as low affinity binding sites while being insensitive to competition from endogenous 5-HT levels [51] and 2) it is still assumed to bind to internalized receptors due to its lipophilic properties [52, 53]. However, this is not to say, that receptor binding is not changed, but potentially not affected to such an extent that would be detectable with the methodology presented in this work. The distinction between outcome measures is especially relevant in light of studies showing alterations in 5-HT_1A_ binding using BP_F_ (ratio of specifically bound radioligand in tissue and free radioligand in plasma) or BP_P_ (ratio of specifically bound radioligand in tissue and total radioligand in plasma) after antidepressant treatment [30, 54]. Hence, even though a possible treatment effect acting on 5-HT_1A_ BP_ND_ cannot be ruled out, we are not convinced that the herein-reported alterations in BP_ND_, specifically in MRN and DRN, primarily reflect a medication effect. However, if contrary to our assumption the signal was mainly driven by a drug effect, then our results would indicate a measurable dissociation between a molecular treatment effect on the one hand and the absence of symptom relief on the other.

Besides lower BP_ND_ of 5-HT_1A_ receptor in subjects with TRD, we found a significant effect of sex on 5-HT_1A_ receptor BP_ND_. However, we could not show significant differences in any ROI specifically (see Supplementary Table 2). Other neuroimaging studies report lower 5-HT_1A_ receptor binding potential in women than in men. Nevertheless, the results are either borderline significant [55] or lacking significance altogether [30, 56]. In contrast, a post-mortem study [57] reported higher serotonin 1A binding in female suicide victims. These findings again highlight the importance of considering the potential effects of sex in study design and analysis [5, 58].

While sex differences were shown within the combined study cohort of healthy control subjects and subjects with TRD, the effect of depressive symptom severity was only investigated in the TRD subject group. We found an impact of symptom severity on 5-HT_1A_ receptor availability, yet post-hoc analyses did not show a significant association of HAMD in any ROI. The lack of a region-wise correlation might be due to an absence of mild or moderate levels of depression, but it is in line with previous research [6, 29]. Unlike symptom severity, age had no significant effect on BP_ND_. Likewise, a study including a large cohort of 61 healthy male subjects [59] found no significant correlation between age and 5-HT_1A_ receptor BP_ND_. However, investigating the effects of sex and age in healthy volunteers [55] reported a tendency towards an inverse relationship between age and postsynaptic 5-HT_1A_ receptor binding potential in men and a small positive association in women.

Finally, some limitations to the study have to be considered. First, the lack of arterial blood samples or population based input function limited our ability to quantify BP_P_ and BP_F_, which were found to be a more accurate estimation of 5-HT_1A_ receptor abundance [29, 30]. Similarly to our results, previous work on MDD patients reports changes in raphe nuclei [6, 9, 28]. While publications investigating MDD cohorts using these outcome measures report changes in DRN as well, the direction of the change in 5-HT_1A_ receptor binding depends on the metric [5, 11, 12]. While the alterations to 5-HT_1A_ receptor are evident in most of the studies, the directionality of these changes is not clear. For example, contrary to the findings of reduced BP_ND_ in depressed patients, studies using BP_F_ which is not available with the reference tissue modelling, showed increased binding [11, 60]. The contrasting outcomes emphasize the role of careful interpretation of presented results. Moreover, an additional limitation arises from the lack of an other than treatment-resistant MDD cohort in the study, which prevents a direct comparison of TRD and MDD subjects. Second, an imbalance in age and sex between the healthy control group and the TRD group might be a limiting factor. Finally, although TRD subjects were on stable medication, the diversity of the accompanying treatment might affect the final result. Due to the variety of concomitant medications (see Supplementary Table 1), such an effect could not be investigated in the current study. In addition, the potential effect of the heterogeneous medication in the medication group is addressed by the harmonization step.

In conclusion, we show a significantly lower 5-HT_1A_ autoreceptor binding in the dorsal and median raphe nuclei of treatment-resistant depressive subjects. Thus, with regard to the ongoing discussion about the role of serotonin in depression, the presented results support the notion of a serotonergic imbalance and extend previous findings in MDD patients to treatment-resistant depression.

## Supplementary information

Supplementary material

## Data Availability

Raw data will not be publicly available due to reasons of data protection. Processed data and custom code can be obtained from the corresponding author with a data-sharing agreement, approved by the departments of legal affairs and data clearing of the Medical University of Vienna.
