# Supplementary material for: In vivo serotonin 1A receptor distribution in treatment-resistant depression
Source: Transl Psychiatry. 2025 Jun 3;15:186. doi: 10.1038/s41398-025-03406-3 (PMC12134062; doi:10.1038/s41398-025-03406-3)
Supplement: Supplementary file 1 — Supplementary material [file 41398_2025_3406_MOESM1_ESM.docx]

**Supplementary information**

***In vivo* serotonin 1A receptor distribution in treatment-resistant depression**

Matej Murgaš MSc. ^1,2*^, Christian Milz MSc. ^1,2*^,

Peter Stöhrmann MSc ^1,2^, Jakob Unterholzner MD ^1,2^, Lukas Nics, PhD ^3^,

Georg S. Kranz, Assoc. Prof. ^4^, Andreas Hahn, Assoc. Prof. ^1,2^, Marcus Hacker, Prof. ^3^, Siegfried Kasper, Prof. ^1,5^, Rupert Lanzenberger Prof. ^1,2#^, Godber M. Godbersen PhD ^1,2#^

(^*^ contributed equally, ^#^ corresponding authors)

*^1^Department of Psychiatry and Psychotherapy, Medical University of Vienna, Vienna, Austria*

*^2^Comprehensive Center for Clinical Neurosciences and Mental Health, Medical University of Vienna, Vienna, Austria*

*^3^Department of Biomedical Imaging and Image-guided Therapy, Division of Nuclear Medicine, Medical University of Vienna, Vienna, Austria*

*^4^Department of Rehabilitation Sciences, The Hong Kong Polytechnic University, Hung Hom, Hong Kong*

*^5^Center for Brain Research, Department of Molecular Neuroscience, Medical University of Vienna, Vienna, Austria.*

| ID | Primary Diagnosis | ICD-10 | SSRI | SNRI | NaSSa | NDRI | ADD | APD | PAM | CB | BZD | EM | OM |
| --- | --- | --- | --- | --- | --- | --- | --- | --- | --- | --- | --- | --- | --- |
| 1 | Recurrent depressive disorder | F33.2 |  | venlafaxine | mirtazapine |  |  |  | zolpidem | lamotrigine | alprazolam |  | lisinopril |
| 2 | Recurrent depressive disorder | F33.2 | citalopram |  | mirtazapine |  |  | prothipendyl | zolpidem |  | lorazepam |  | aliskiren, esomeprazole, amlodipin, metoprolol |
| 3 | Recurrent depressive disorder | F33.2 |  |  | mirtazapine |  |  | prothipendyl |  |  | lorazepam |  | estradiol, dydrogesterone, omeprazole |
| 4 | Recurrent depressive disorder | F33.2 |  | venlafaxine |  |  |  | prothipendyl amisulpride | zolpidem |  | lorazepam |  | lisinopril, esomeprazol, folic acid |
| 5 | Recurrent depressive disorder | F33.2 | sertraline | venlafaxine |  |  |  |  |  |  | lorazepam |  | levothyroxine |
| 6 | Recurrent depressive disorder | F33.2 |  | milnacipran |  |  |  | prothipendyl |  | lamotrigine oxcarbazepine | lorazepam |  | omeprazole, beta-blocker, hydrochlorothiazide, tiotropium bromide |
| 7 | Recurrent depressive disorder | F33.2 |  | duloxetine | mirtazapine |  |  | prothipendyl amisulpride |  |  |  | lithiumcarbonat |  |
| 8 | Recurrent depressive disorder | F33.2 |  | duloxetine | mirtazapine |  |  |  |  | pregabalin |  |  |  |
| 9 | Recurrent depressive disorder | F33.2 | fluoxetine |  |  |  |  |  |  |  | lorazepam, triazolam |  | esomeprazol |
| 10 | Recurrent depressive disorder | F33.2 | escitalopram |  |  | bupropion |  | prothipendyl |  |  | lorazepam, alprazolam |  | metformin, gliclazide |
| 11 | Recurrent depressive disorder | F33.2 | sertraline |  |  |  |  | prothipendyl |  |  | lorazepam |  | pantoprazole, mebeverine hydrochloride, loperamide |
| 12 | Recurrent depressive disorder | F33.2 |  | duloxetine |  | bupropion |  |  |  |  |  |  | bisoprolol, lisinopril |
| 13 | Recurrent depressive disorder | F33.2 | fluoxetine |  | mirtazapine |  |  | olanzapine | zolpidem |  |  | lithiumcarbonat |  |
| 14 | Recurrent depressive disorder | F33.2 |  | duloxetine |  |  |  |  |  |  |  |  | pantoprazole |
| 15 | Recurrent depressive disorder | F33.1 |  |  |  |  |  | quetiapine |  |  |  |  |  |
| 16 | Recurrent depressive disorder | F33.2 | sertraline escitalopram |  |  |  |  |  |  |  |  |  |  |
| 17 | Major depressive episode | F32.1 |  | venlafaxine |  |  |  |  |  |  |  |  |  |
| 18 | Recurrent depressive disorder | F33.2 |  |  |  |  | melitracen | flupentixole |  | pregabalin |  |  |  |
| 19 | Recurrent depressive disorder | F33.1 | sertraline |  |  |  |  |  |  | pregabalin |  |  |  |
| 20 | Recurrent depressive disorder | F33.2 | escitalopram |  |  |  |  | quetiapine |  | pregabalin |  | lithiumcarbonat | beta-blocker |
| 21 | Recurrent depressive disorder | F33.1 |  | milnacipran |  |  | melitracen | prothipendyl flupentixole |  |  | alprazolam |  |  |
| 22 | Recurrent depressive disorder | F33.2 |  | venlafaxine |  |  |  |  | zolpidem |  | lorazepam |  | pantoprazole, beta-blocker |
| 23 | Recurrent depressive disorder | F33.1 | escitalopram |  |  | bupropion |  |  |  |  |  |  |  |
| 24 | Recurrent depressive disorder | F33.1 |  | venlafaxine |  | bupropion |  |  |  |  |  |  |  |
| 25 | Recurrent depressive disorder | F33.1 | sertraline |  |  |  |  |  |  |  |  |  |  |
| 26 | Recurrent depressive disorder | F33.1 | escitalopram |  |  |  | mianserin |  |  |  |  |  |  |
| 27 | Recurrent depressive disorder | F33.1 | escitalopram |  |  |  |  |  |  |  |  |  |  |
| 28 | Recurrent depressive disorder | F33.1 |  | venlafaxine |  |  |  |  |  |  |  | lithiumcarbonat | candesartan, cilexetil,  beta-blocker, levothyroxine |
| 29 | Recurrent depressive disorder | F33.1 | citalopram |  |  |  | tizanidine |  | zolpidem |  |  |  | pantoprazole |
| 30 | Recurrent depressive disorder | F33.1 | escitalopram | milnacipran | mirtazapine |  |  |  |  |  |  |  |  |
| 31 | Recurrent depressive disorder | F33.1 |  |  |  |  | reboxetine melitracen | flupentixole |  |  |  |  |  |
| 32 | Recurrent depressive disorder | F33.1 | sertraline | venlafaxine |  |  |  |  |  |  |  |  |  |
| 33 | Recurrent depressive disorder | F33.1 |  | venlafaxine |  |  |  |  |  |  |  |  |  |

**Supplementary Table 1: List of concomitant treatment in TRD patients with the details on the diagnosis;** SSRI - selective serotonin reuptake inhibitor, SNRI - serotonin-noradrenaline reuptake inhibitor, NaSSa - noradrenergic and specific serotonergic antidepressant, NDRI - norepinephrine–dopamine reuptake inhibitor, ADD - other antidepressant drugs, APD - antipsychotic drug, PAM - positive allosteric modulator, CB - channel blocker, BZD - benzodiazepine, EM - enzyme modulator, OM - Other medication. ICD-10 codes: F32.1 Moderate depressive episode; F33.1 Recurrent depressive disorder, current episode moderate; F33.2 Recurrent depressive disorder, current episode severe without psychotic symptoms

|  | **EMM of 5-HT_1A_ receptor BP_ND_** | | | **Pairwise Comparisons** | | | | |
| --- | --- | --- | --- | --- | --- | --- | --- | --- |
| **ROI** | **F** | **M** | **PD[%]** | **mean diff.** | **CI (95%)** | **Sig.** | **η^2^** |  |
| ACC | 4.76 ± 1.02 | 4.59 ± 1.12 | 3.91 | 0.17 ± 0.24 | (-0.66, 0.31) | 0.48 | 0.007 |  |
| AMY | 3.78 ± 0.85 | 3.94 ± 0.93 | -3.84 | -0.16 ± 0.20 | (-0.25, 0.56) | 0.44 | 0.008 |  |
| HIP | 5.57 ± 1.38 | 5.87 ± 1.52 | -5.05 | -0.30 ± 0.33 | (-0.74, 0.61) | 0.37 | 0.011 |  |
| INS | 6.71 ± 1.42 | 6.65 ± 1.57 | 1.35 | 0.06 ± 0.34 | (-0.36, 0.95) | 0.85 | <0.001 |  |
| OFC | 4.85 ± 0.98 | 4.62 ± 1.08 | 5.26 | 0.23 ± 0.23 | (-0.69, 0.24) | 0.33 | 0.013 |  |
| DRN | 2.95 ± 0.74 | 3.19 ± 0.82 | -7.00 | -0.25 ± 0.18 | (-0.11, 0.60) | 0.17 | 0.026 |  |
| MRN | 3.44 ± 0.91 | 3.52 ± 1.00 | -1.46 | -0.08 ± 0.22 | (-0.35, 0.51) | 0.72 | 0.002 |  |

**Supplementary Table 2: Comparison of 5-HT_1A_ BP_ND_ between male and female subjects.** Estimated marginal means (EMM) and pairwise comparison of 5-HT_1A_ receptor BP_ND_ between female (F) and male (M) groups including whole subject cohort (healthy control subjects and patient) anterior cingulate cortex (ACC), amygdala (AMY), hippocampus (HIP), insula (INS), orbitofrontal cortex (OFC), dorsal raphe nucleus (DRN) and median raphe nucleus (MRN). Marginal means are given as mean ± standard deviation and are corrected for sex and age. Percent difference (PD) representing change of BP_ND_ in the individual regions is calculated as ${PD=\left[ {BP}_{ND}\left( F \right) - {BP}_{ND}\left( M \right) \right]}/{{BP}_{ND}\left( M \right)\cdot100}\%$ with η^2^ representing the estimated effect size*.* An exploratory post-hoc pairwise comparison is reported as the mean difference (± standard error) between the marginal mean ${BP}_{ND}\left( F \right)$ and ${BP}_{ND}\left( M \right)$ with 95% confidence intervals (CI) and η^2^ representing the estimated effect size. The difference in marginal means was considered significant when p<0.05.


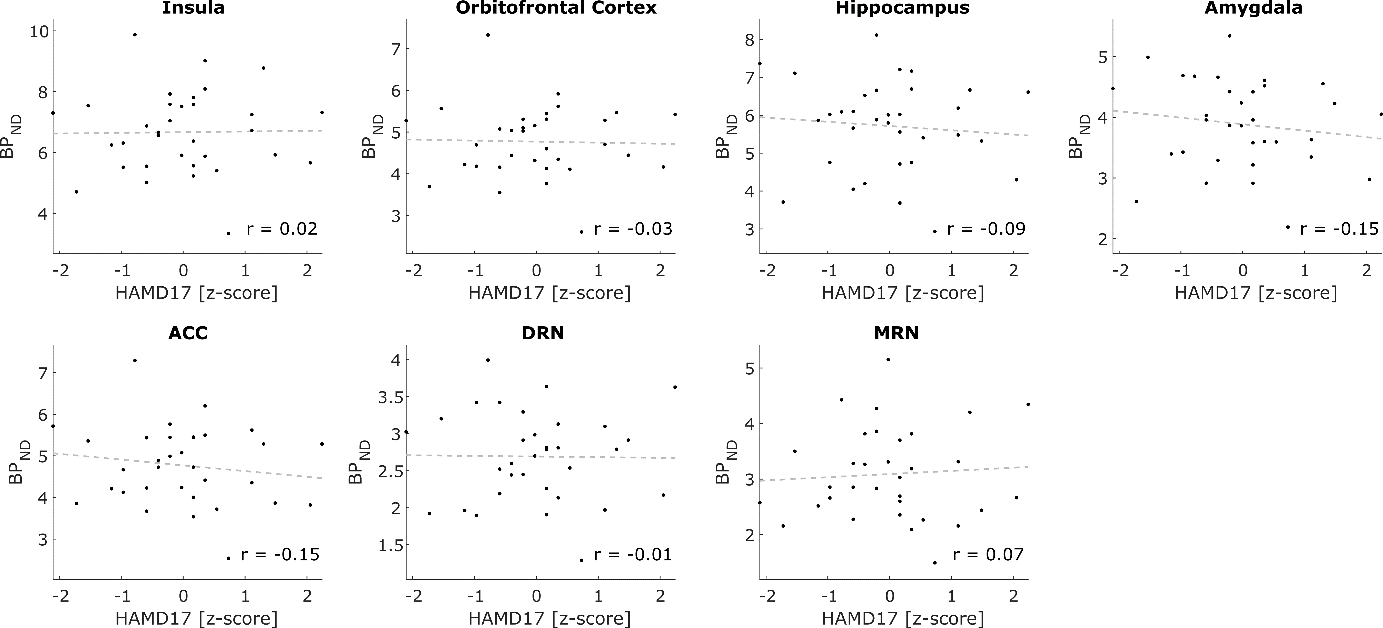


**Supplementary Figure 1: Association between the symptom severity and 5-HT_1A_ receptor binding.** Scatter plots with reference line showing the statistically non-significant (p>0.05) relation between HAMD score (z-score) and 5-HT_1A_ receptor binding in insula, orbitofrontal cortex, hippocampus, amygdala, anterior cingulate cortex (ACC), dorsal (DRN) and median (MRN) raphe nuclei. The Pearson correlation coefficient (r) is shown in the left bottom corner of each scatter plot.
